# Effectiveness of a Single Intra-articular Injection of High-Molecular-Weight Hyaluronic Acid Versus NSAIDs on the Visual Analog Pain Scale and Western Ontario and McMaster Universities Osteoarthritis Index Scores in Patients With Knee Osteoarthritis

**DOI:** 10.7759/cureus.69313

**Published:** 2024-09-13

**Authors:** Suriya Shahaly, Mohammad Moniruzzaman, Nusrat Neherin Khan, Md Iftakharul Alam, Sayat Quayum, Shahina Sarker, Humayun Kabir Sarker, Md Muhibbur Rahman

**Affiliations:** 1 Department of Physical Medicine and Rehabilitation, Ahsania Mission Cancer and General Hospital, Dhaka, BGD; 2 Department of Physical Medicine and Rehabilitation, Dhaka Medical College and Hospital, Dhaka, BGD; 3 Department of Medicine, International Centre for Diarrhoeal Diseases Research, Bangladesh, Dhaka, BGD; 4 Department of Physical Medicine and Rehabilitation, Parkview Medical College Hospital, Sylhet, BGD; 5 Department of Internal Medicine, Evercare Hospital, Dhaka, BGD; 6 Department of Physical Medicine and Rehabilitation, Savar Upazila Health Complex, Dhaka, BGD; 7 Department of Interventional Neurology, National Institute of Neurosciences, Dhaka, BGD; 8 Department of Physical Medicine and Rehabilitation, Sarkari Karmachari Hospital, Dhaka, BGD

**Keywords:** high-molecular-weight hyaluronic acid, knee osteoarthritis, nonsteroidal anti-inflammatory drugs, single intra-articular injection, visual analogue pain scale, womac score

## Abstract

Objective: This study aimed to determine the effectiveness of a single intra-articular injection of high-molecular-weight (HMW) hyaluronic acid (HA) at a dose of 4 mL/60 mg to reduce pain in people with knee osteoarthritis (OA) over 12 months.

Methodology: This retrospective study was conducted after obtaining ethical approval from Dhaka Medical College and Hospital, Bangladesh. From July 2020 to June 2021, a medical professional conducted the investigation. The investigation encompassed patients aged 40 to 70 hospitalized at our facility and diagnosed with Grade 2 or Grade 3 knee OA according to the Kellgren-Lawrence grading method. We divided the patients into two categories based on the treatments they received. Patients in Group A received a single injection of HMW HA (60 mg/4 mL) into the joint, along with instructions on activities of daily living, exercise, and painkillers. Group B patients received conservative therapy, which involved the use of nonsteroidal anti-inflammatory drugs (NSAIDs), muscle relaxants, therapeutic exercises, and a knee brace during physical activity.

Results: This study compared the efficacy of a single injection of HMW HA in the joint versus NSAIDs for managing OA-related symptoms over 12 weeks. The HA group initially scored similarly to the comparison group. By week three, however, the group receiving HA had considerably higher Western Ontario and McMaster Universities Osteoarthritis Index (WOMAC) scores (p=0.019). This pattern continued through weeks 6 (p=0.044), 9 (p=0.016), and 12 (p<0.001). Similarly, by week 3 (p=0.029), the visual analog scale (VAS) scores, which were initially identical (p=0.120), demonstrated a significant preference for HA, and this preference persisted through weeks 6, 9, and 12 (all p<0.001). The results show that by the third week, HA is more effective than NSAIDs at relieving pain and improving symptoms.

Conclusion: The study’s results indicated that over 12 weeks, the use of HMW HA led to statistically significant reductions in pain intensity, as measured by the VAS. Furthermore, HMW HA demonstrated a more significant improvement in WOMAC ratings, which assess physical function, stiffness, and pain, compared to NSAIDs. The findings suggest that administering HMW HA injections can significantly reduce symptoms and improve functionality in individuals with knee OA.

## Introduction

Knee osteoarthritis (OA) is a prevalent and progressive condition that predominantly impacts the joints and is diagnosed in a significant number of individuals worldwide, with an estimated 250 million affected [1]. Globally, knee and hip OA is the 11th most prevalent disability, increasing with age [2,3]. The symptoms of this degenerative illness are characterized by inflammation and cartilage degeneration, which impede daily activities and reduce overall quality of life. Pain, rigidity, and diminished functional capabilities are among these symptoms [4]. Additionally, these symptoms have a substantial effect on healthcare expenditures. Nonsteroidal anti-inflammatory drugs (NSAIDs), alternative painkillers, corticosteroids injected into the affected joint, localized therapy, hyaluronic acid (HA), and surgery are common treatments for OA [5]. Despite the infrequent use of physical agent modalities in OA treatment, the lack of compelling evidence has hindered their effectiveness [6]. Recent research has revealed botulinum toxin intra-articular injections as a potential treatment for pain in the early stages of OA, demonstrating optimistic results [4]. In recent years, intra-articular injections of corticosteroids, ozone, HA, and platelet-rich plasma have become more popular as alternatives to NSAIDs [7,8]. This shift aims to reduce adverse effects and expedite pain relief [9].

The findings of a Boyer study [10] show that intra-articular HA injections, a typical treatment procedure, offer many advantages and have reduced the need for analgesic medication. HA, a polymer found in cartilage and synovial fluid, is considered one of the most essential polysaccharides. Its joint benefits include stress absorption, lubrication, and cartilage preservation [11]. Researchers have determined that inflammation is the principal source of the breakdown and excretion of a considerable amount of endogenous synovial HA in OA [12]. Furthermore, HA's biological effects can differ based on its molecular weight, concentration, source, dosage, cross-linkage, and formulation [13]. Recent randomized clinical trials have demonstrated that using an external form of HA (viscosupplementation) improves the treatment of knee OA by relieving symptoms [11,14].

HA formulations for intra-articular use come in different molecular weights. Low-molecular-weight preparations (0.25-1 million daltons) result in greater joint concentrations and have the potential to reduce inflammation; however, compared to natural HA, they are less elastoviscous [15]. Preparations with larger molecules, having molecular weights of 2.7-7 million daltons, may improve joint fluid retention and have more potent anti-inflammatory effects [16].

The efficacy of HA treatment varies depending on the formulation’s rheological properties and molecular weight [17]. Recent studies on using HA with different molecular weights to treat knee OA have yielded conflicting results. However, there appears to be a tendency to use high-molecular-weight (HMW) HA [18,19]. Despite numerous studies demonstrating the effectiveness of a single HA injection, research directly comparing the benefits of a single HA injection to multiple HA injections in individuals with OA is scarce. Furthermore, most clinical trials evaluating HA injections have observation durations of approximately six months [20]. This study aimed to investigate the efficacy of a single intra-articular injection of HMW HA at a dosage of 4 mL/60 mg in reducing pain in individuals with knee OA over one year.

## Materials and methods

This retrospective study was conducted after obtaining ethical approval from Dhaka Medical College and Hospital, Bangladesh, under DMCH/312/PHD/2020. From July 2020 to June 2021, a medical professional conducted the investigation. The study included patients aged 50 to 60 hospitalized at our facility and diagnosed with Grade 2 or Grade 3 knee OA according to the Kellgren-Lawrence grading method. The symptoms of knee OA had been present for 12 to 18 months in these patients. Patients with a history of knee surgery, a BMI greater than 30, neoplasia, cognitive impairment, Grade 1 or Grade 4 knee OA, or prior implantation in the same limb were excluded.

Patients were divided into two groups based on the treatments they received. Group A patients received a single intra-articular injection of HMW HA (60 mg/4 ml), along with instructions for activities of daily living (ADLs), exercise, and analgesics. They were placed in a supine position with their knees fully extended [18]. The HA was injected into the supra-patellar pouch using a pre-filled syringe containing 3 ml of local anesthetic (2% lidocaine) under stringent aseptic conditions and through a supra-lateral approach. After the injection, patients were advised to apply ice to the affected area and rest for one day. From the second day onward, they were allowed to resume their normal activities and were instructed to take paracetamol for pain relief. Group B patients received conservative therapy, which included NSAIDs, muscle relaxants, therapeutic exercises, and a knee brace during physical activities. They also received guidelines for ADLs. The exercise regimen included strengthening the quadriceps, particularly the vastus medialis oblique, and activities to extend and strengthen the hamstrings. Quadriceps strengthening involved knee extension exercises, and to fortify the vastus medialis oblique, patients performed isometric hip adduction exercises while squatting. For hamstring exercises, patients stretched their legs while lying on their backs with a towel, holding the extended position for five seconds, followed by a 10-second rest. Additionally, all patients were instructed on ADLs and advised to use paracetamol for pain relief.

The variables of interest were overall satisfaction, functional status, and pain levels. Data from the medical records of both groups were used to compare these variables. Clinical parameters, including the Western Ontario and McMaster Universities Osteoarthritis Index (WOMAC) and the visual analog scale (VAS), were recorded during follow-up visits at one month, three months, six months, and 12 months after the most recent intra-articular injection. These parameters were used to assess patient function and pain. The WOMAC is a validated tool widely used to assess pain, stiffness, and physical function in OA patients. WOMAC is also responsive to clinical changes, making it suitable for evaluating treatment outcomes.

Statistical analysis was conducted using SPSS Statistics version 22.00 (IBM Corp. Released 2013. IBM SPSS Statistics for Windows, Version 22.0. Armonk, NY: IBM Corp.). The normality of the data was evaluated using the Shapiro-Wilk test. Differences between categorical variables were investigated using the chi-square test. Mean values within each group were compared using the paired t-test, while differences between groups were analyzed using the independent t-test. Statistical significance was set at a p-value of less than 0.05.

## Results

The mean age of the participants in the HA group was 56.9 ± 9.7 years, whereas the NSAID group had a mean age of 53.1 ± 7.2 years. The non-significant p-value of 0.149 suggests no substantial variation in age between the two groups. The HA group had a sex distribution of 6 (20.7%) male and 23 (79.3%) female, whereas the NSAID group had a sex distribution of 11 (42.3%) male and 15 (57.7%) female. The p-value of 0.09 indicates no statistically significant difference in sex between the two groups. We employed the Kellgren-Lawrence grade to evaluate the extent of OA. Of the participants, 28 (96.6%) in the HA group were classified as Grade 2, while one (3.4%) was classified as Grade 3. On the other hand, 24 (92.3%) of individuals in the NSAID group were classified as Grade 2 and 2 (7.7%) as Grade 3. The p-value of 0.578 indicates no statistically significant difference between the two groups. The distribution of symptoms was as follows: in the HA group, 15 (51.7%) of participants experienced symptoms on the left side and 14 (48.3%) on the right side, while in the NSAID group, nine (34.6%) experienced symptoms on the left side and 17 (65.4%) on the right side. The p-value of 0.203 indicates that this difference is not statistically significant. The study’s findings indicate that the initial characteristics of the groups were similar, implying a fair comparison in assessing the effectiveness of the treatments. Table 1 shows the characteristics of both groups.

**Table 1:** Characteristics of both groups * indicates t-test value, ** indicates chi-square values HMW: high molecular weight, HA: hyaluronic acid, NSAID: nonsteroidal anti-inflammatory drug, SD: standard deviation

Parameters	Single intra-articular injection of HMW HA	NSAID group	Statistical test value	p-value
Age in years (mean ± SD)	56.9 ± 9.7	53.1 ± 7.2	1.634*	0.149
Sex			2.073**	0.09
Male	6 (20.7%)	11 (42.3%)		
Female	23 (79.3%)	15 (57.3%)		
Kellgren-Lawrence grade			0.0009**	0.578
Grade 3	1 (3.4%)	2 (7.7%)		
Grade 2	28 (96.6%)	24 (92.3%		
Side of involvement			1.79**	0.203
Left	15 (51.7%)	9 (34.6%)		
Right	14 (48.3%)	17 (65.4%)		

Table 2 displays the findings of a study conducted on a group of individuals who were administered NSAIDs. The study aimed to assess pain intensity over 12 weeks using the VAS. In week 0, the average VAS score was 8 ± 0.5. By the third week, the average VAS score had decreased to 6.8 ± 0.7. The trend of decline continued in weeks 6 (6 ± 1), 9 (5.4 ± 1.3), and 12 (4.8 ± 1.6). For all instances, the p-values were less than 0.001, indicating that the declines in VAS scores from week 0 to each subsequent time point were significant. This suggests a consistent and significant decrease in pain during the 12 weeks.

**Table 2:** Comparison of VAS score of NSAID from baseline to week 12 VAS: visual analog scale, NSAID: nonsteroidal anti-inflammatory drug, SD: standard deviation

VAS score	Mean ± SD	Paired t-test value	p-value
At week 0	8 ± 0.5	0	<0.001
At week 3	6.8 ± 0.7	6.32	<0.001
At week 6	6 ± 1	10.53	<0.001
At week 9	5.4 ± 1.3	13.68	<0.001
At week 12	4.8 ± 1.6	16.84	<0.001

The baseline VAS score for the HA injection group was 8.1 ± 0.6, measured due to a single intra-articular injection of HMW HA at week 0. By the third week, the average VAS score had decreased to 6.2 ± 0.8. In week 6, the average score was 4.8 ± 1.1, which continued to decrease until week 9, when it was 4.1 ± 1.3. The average VAS score at week 12 was 2.8 ± 1.4. The p-values for these reductions were all less than 0.0001, indicating that the decreases in pain intensity from week 0 to each subsequent time point were highly statistically significant, demonstrating a continuous and significant decrease in pain throughout the study (Table 3).

**Table 3:** Comparison of VAS score of HA injection from baseline to week 12 VAS: visual analog scale, HA: hyaluronic acid, SD: standard deviation

VAS score	Mean ± SD	Paired t-test value	p-value
At week 0	8.1 ± 0.6	0	<0.0001
At week 3	6.2 ± 0.8	7.92	<0.0001
At week 6	4.8 ± 1.1	13.75	<0.0001
At week 9	4.1 ± 1.3	16	<0.0001
At week 12	2.8 ± 1.4	18.93	<0.0001

The study, conducted over 12 weeks, aimed to assess the comparative efficacy of a single intra-articular injection of HMW HA and NSAIDs in reducing pain. We utilized the VAS to achieve this purpose. Initially (week 0), the average VAS scores were similar: 8.1 ± 0.6 for the HA group and 8 ± 0.5 for the NSAID group, with a p-value of 0.120 suggesting no significant difference. By the third week, the group that received HA treatment experienced a decrease in their score to 6.2 ± 0.8, whereas the group treated with NSAIDs scored 6.8 ± 0.7. The p-value of 0.029 suggests a statistically significant difference between the two groups. This trend continued, as shown by the substantial differences detected at week 6 (4.8 ± 1.1 vs. 6 ± 1, p<0.001), week 9 (4.1 ± 1.3 vs. 5.4 ± 1.3, p<0.001), and week 12 (2.8 ± 1.4 vs. 4.8 ± 1.6, p<0.001). The data suggest that the use of HA injection led to a greater and statistically significant decrease in pain compared to NSAIDs, especially during the third week (Table 4).

**Table 4:** Comparison of VAS score of HA injection vs. NSAID group VAS: visual analog scale, HMW: high molecular weight, HA: hyaluronic acid, NSAID: nonsteroidal anti-inflammatory drug, SD: standard deviation

VAS score	Single intra-articular injection of HMW HA mean ± SD	NSAID group mean ± SD	t-test value	p-value
At week 0	8.1 ± 0.6	8 ± 0.5	0.69	0.120
At week 3	6.2 ± 0.8	6.8 ± 0.7	-3.09	0.029
At week 6	4.8 ± 1.1	6 ± 1	-4.41	<0.001
At week 9	4.1 ± 1.3	5.4 ± 1.3	-3.87	<0.001
At week 12	2.8 ± 1.4	4.8 ± 1.6	-5.19	<0.001

The study contrasted the efficacy of a single intra-articular injection of HMW HA and NSAIDs in improving WOMAC scores, which assess pain, rigidity, and physical function in OA over 12 weeks. The NSAID group scored 80.2 ± 6.2, whereas the HA group scored 77.9 ± 6.4 (p=0.334). At the outset, the WOMAC scores of both groups were comparable. In week 3, the HA group’s score increased substantially to 71.3 ± 6.7, while the NSAID group’s score remained at 74.9 ± 4.8 (p=0.019). The trend persisted at weeks 6, 9, and 12, with scores of 68.3 ± 7.2 vs. 72.5 ± 4.9 (p=0.044), 66 ± 7.1 vs. 70.2 ± 4.9 (p=0.016), and 61.7 ± 6.5 vs. 68.2 ± 4.5 (p<0.001), respectively. The HA injection resulted in more substantial and statistically significant improvements in WOMAC scores than NSAIDs, particularly in the third week, as indicated by the data (Table 5).

**Table 5:** Comparison of WOMAC score of HA injection vs. NSAID group WOMAC: Western Ontario and McMaster Universities Osteoarthritis Index, HMW: high molecular weight, HA: hyaluronic acid, NSAID: nonsteroidal anti-inflammatory drug, SD: standard deviation

WOMAC score	Single intra-articular injection of HMW HA mean ± SD	NSAID group mean ± SD	t-test value	p-value
At week 0	77.9 ± 6.4	80.2 ± 6.2	-1.41	0.334
At week 3	71.3 ± 6.7	74.9 ± 4.8	-2.40	0.019
At week 6	68.3 ± 7.2	72.5 ± 4.9	-2.63	0.044
At week 9	66 ± 7.1	70.2 ± 4.9	-2.67	0.016
At week 12	61.7 ± 6.5	68.2 ± 4.5	-4.52	<0.001

## Discussion

This study aimed to evaluate the effectiveness of a single intra-articular injection of HMW HA at a dosage of 2 mL/60 mg in persons with knee OA. The study analyzed pain scores over 12 months. Our research revealed a significant reduction in pain on the VAS among participants in the HA group. Following a three-week HMW HA treatment, the VAS scores were reduced from 8.3 ± 0.7 to 6.3 ± 0.9. The VAS score decreased with each follow-up session. During the 12th week, the VAS ratings were reduced to 2.8 ± 1.3, indicating a notable deviation from the initial baseline readings. Therefore, it can be inferred that utilizing HMW HA during the 12-week follow-up period leads to a statistically significant decrease in VAS scores. OA leads to a reduction in both the quantity and molecular weight of HA in synovial fluid. The goal of injecting HA into the joint is to reinstate the synovial fluid’s viscoelastic properties. Injecting external HA enhances synovial fluid flow, controls the production of HA in the body, and reduces joint discomfort [21]. Our investigation revealed a notable reduction in the VAS among participants in the NSAID group, with scores decreasing from 8.0 ± 0.5 to 6.9 ± 0.8 after three weeks of conservative treatment. The VAS score fell with each follow-up session. During the 12th week, the VAS ratings were reduced to 4.9 ± 1.7, indicating a notable deviation from the initial baseline readings.

According to our research, WOMAC scores dropped significantly after treatment with HMW HA. They went from an initial score of 78.1 ± 6.3 to 71.2 ± 6.6 three weeks later. The WOMAC score consistently decreased throughout each follow-up period. In the 12th week, the WOMAC scores were reduced to 61.9 ± 6.6, showing a statistically significant reduction compared to the initial baseline. Research has demonstrated that HMW HA hinders the generation of proinflammatory cytokines, such as interleukin-1α, interleukin-6, and tumor necrosis factor-α. It has greater efficacy in avoiding the reduction of proteoglycan (PG) levels in damaged cartilage and restoring PG content [11].

A recent clinical investigation has shown that HA viscosupplementation has anti-inflammatory and chondroprotective effects, reducing pain and improving patient functionality. The duration of these effects can vary between four and 14 weeks [9]. Meanwhile, in Group B, there was a notable reduction in WOMAC scores from the initial evaluation of 80.1 ± 6.1 to 75.0 ± 4.9 at the three-week mark following conservative management treatment. The WOMAC score consistently decreased throughout each follow-up period. In the 12th week, the WOMAC scores were reduced to 68.1 ± 4.6, showing a statistically significant reduction compared to the initial baseline. However, there was no significant difference in the VAS scores between the two groups. Following a six-week treatment, the average VAS scores of the patients in Group A and Group B were 4.9 ± 1.1 and 6.0 ± 1.0, respectively. The difference in scores between the two groups showed statistical significance. After 12 weeks, the VAS score demonstrated a noteworthy decrease in Group A (2.8 ± 1.3) compared to Group B (4.9 ± 1.7).

Dougados et al. [22] conducted a study that found providing HMW HA through intra-articular injections can enhance the clinical condition and provide long-term benefits to patients with knee OA. Wu et al. [23] found that HMW HA significantly reduced VAS scores. The patients in Group A of our study had a baseline WOMAC score of 78.1 ± 6.3, while in Group B, the mean WOMAC score was 80.1 ± 6.1. There was no significant difference in WOMAC scores between the two groups, as indicated by a p-value of 0.304. Following a three-week intervention, the average WOMAC scores of the patients in Group A and Group B were 71.2 ± 6.6 and 75.0 ± 4.9, respectively. The difference in scores between the two groups was statistically significant. After 12 weeks, the WOMAC score demonstrated a noteworthy decrease in Group A (61.9 ± 6.6) compared to Group B (68.1 ± 4.6). HMW HA exhibits remarkable effectiveness [24].

Several recent studies have compared the efficacy of various molecular weights of HA. Wu et al.'s [23] meta-analysis scrutinized numerous studies. It determined that injections of HMW HA yielded the greatest benefits for six months following therapy without any additional risk of negative consequences [23]. Concoff et al.'s meta-analysis found that administering intra-articular injections of HA to patients with knee OA resulted in more substantial pain alleviation compared to intra-articular saline [25]. The treatment regimen consisted of a range of two to four shots. The findings of our investigation provide evidence for HMW HA’s effectiveness, as demonstrated by both single and repeated administrations. In 2009, Chevalier et al. [26] undertook a six-month study to assess the efficacy of different doses of Hylan G-F 20 (Synvisc) in 120 individuals suffering from knee OA. The study used a randomized and multicenter approach. They categorized their participants into five groups: one group received a 6-milliliter injection, another group received a 4-milliliter infusion, and the remaining groups received a combination of dosages administered at intervals of two or three weeks. The study’s results showed that administering 2 mL injections once a week for three weeks was less effective or well tolerated compared to a single 6-mL injection. Furthermore, the group that received a single 6-mL injection had the fewest patients needing additional treatment. The trial utilized an FDA-approved dosage for the single-injection formulation. Despite its small sample size and lack of double-blinding, the study demonstrated comparable results in pain relief and functional improvement to the repeated injection series. Saibaba et al [27] compared the efficacy of three different therapies for knee OA. These treatments comprised Hylan G-F 20 (Synvisc) in three injections, HA 60 mg/3 mL (Durolane) in a single injection, and HA 40 mg/2 mL (Osteonil) in three or five injections. The primary goal of their research was to determine the level of variance in WOMAC scores over six months. The study found that Hylan G-F 20 (Synvisc) was the most successful of the three treatments. The medicine significantly improved WOMAC scores compared to HA 40 mg/2 mL (Osteonil) and HA 60 mg/3 mL (Durolane). Furthermore, their study showed that HA 40 mg/2 mL (Osteonil) outperformed HA 60 mg/3 mL (Durolane)

Limitations

Our study has certain limitations. The comparatively small sample size may restrict its applicability, particularly among diverse demographic groups or varying disease severity. The study’s retrospective analysis design introduces potential biases and limits its ability to control for confounding factors completely. Furthermore, the 12-week follow-up period may not adequately evaluate the long-term effects or sustainability of therapy outcomes beyond this specific time frame. To better understand the effectiveness of HMW HA injections over time compared to other treatments for knee OA, extensive, randomized controlled trials with more extended follow-up periods should be conducted in the future. These improvements would fortify the existing corpus of data and offer clinical decision-makers guidance on the most effective method of managing knee OA.

## Conclusions

The study’s results indicated that using HMW HA led to statistically significant reductions in pain intensity, as measured by the VAS, over 12 weeks. Furthermore, HMW HA demonstrated a more significant improvement in WOMAC ratings, which assess physical function, stiffness, and pain, compared to NSAIDs. The findings suggest that administering HMW HA injections can significantly reduce symptoms and improve functionality in individuals with knee OA.

Future research should focus on conducting larger, multi-center trials to validate these findings and evaluate the long-term efficacy and safety of HMW HA across different populations. It would also be beneficial to explore the mechanisms behind HMW HA’s therapeutic effects and compare them with other emerging treatments to refine management strategies for knee OA. Additionally, examining patient-reported outcomes and quality-of-life measures will be essential for fully understanding the benefits of HMW HA therapy.
